# The Julian Assange case and its implications for expert witness evidence

**DOI:** 10.1177/00258024251328790

**Published:** 2025-04-29

**Authors:** Michael D Kopelman

**Affiliations:** 1 King's College London and Forensic Psychiatry Chambers, UK

**Keywords:** Assange, expert witness, professional roles/responsibilities, re-litigation, professional ethos, expert recruitment

## Abstract

The recent Julian Assange case raised a number of important issues regarding the role of expert witnesses in court. While written from a personal perspective, this paper will suggest that these issues need much fuller discussion than they have received to date. They will be discussed in the context of what actually happened in this case, the details of which were reported only sketchily (and sometimes inaccurately) in the press. First, there is the question of what is properly a medical or a legal responsibility. A second issue concerns whether re-litigation of already determined matters should be permitted in higher courts, when the expert does not have the opportunity to respond. A third matter involves the apparently differing professional views and ethos of the legal and other professions regarding matters of personal privacy for non-participants, particularly with respect to the safeguarding of children. Other issues include the language which may be used by some lawyers in criticising expert testimony, the protection of experts from potentially libellous reporting in the press, and the use and abuse of diagnostic classifications, such as the International Classification of Diseases (ICD) and the Diagnostic and Statistical Manual (DSM). These various matters have implications for larger concerns regarding the recruitment of suitable expert witnesses to the courts.

## 1. Introduction

A number of issues arose from the USA v. Julian Assange case concerning the role of experts in legal cases. I myself have long been of the view that we need a larger pool of medical and scientific experts available to the Courts, and that it is highly desirable to encourage leading scientists to provide evidence for the Criminal Courts. In a paper published in 2013, I wrote ‘…A much more important and major issue at the moment is how we get existing clinical science properly represented in the Courts – not the clinical science of the future, but already established clinical science. Everything that is happening to Legal Aid, in the NHS, and in our Universities, is going to make this increasingly problematical’ (Kopelman, 2013).^1^

While the intention of this paper is to draw attention to general issues, which need fuller discussion than they usually receive, it is inevitably based on my experience in this particular case. While there has been much scrutiny of the performance of expert witnesses in recent Judgments and the medico-legal literature (Eastman and Rix, 2022^2^), issues for the legal profession also arise, as will be discussed below.

My own credentials for this case were that I had produced psychiatric reports for a number of high-profile extradition cases over the years (including *Tollman*,^3^ *Norris*,^4^ *Dewani*^5^ and *Love*^6^), receiving approving comments on previous reports from past Lord Chief Justices, including the (then) Sir Thomas Bingham and Sir John Thomas. Indeed, Lord Burnett of Maldon accepted my evidence when Lord Chief Justice in the case of *USA v. Lauri Love*.^7,8^

## 2. The issue of anonymity

As will be known by those who have followed the Julian Assange case, an issue arose concerning the identification of Mr Assange's partner, Stella Moris, and young children. Their existence is now very well known, following the very many interviews and television broadcasts given by Ms Moris, the couple's widely publicised marriage, and Mr Assange's eventual release. However, when I interviewed Mr Assange in Belmarsh prison on a series of occasions in 2019, only a very few people knew of their relationship, which had commenced in the Ecuadorian embassy. When interviewed, both Mr Assange and Ms Moris were extremely concerned to preserve her anonymity and that of her infant children for specific security reasons. Threats had been made to Mr Assange and his family. He had been spied upon in the Ecuadorian embassy. A plot to steal a nappy for the purpose of identifying DNA had been revealed. Confirmation of all this subsequently emerged in the Spanish Courts, and was not contradicted in the English courts, but, in 2019, only a very few people knew of this relationship and the two children (conceived in the embassy). Both Mr Assange and Ms Moris told me about the security threats. I did not know what to make of these security threats (whether they were true or not), and I sought advice from an instructing lawyer,^9^ in which I asked what (given the context) I could or should say in my report (scheduled for December 2019) about the relationship. I followed the advice given, which was, in essence, to make known that a supportive partner and children existed, but that, for the time-being (i.e. pending a decision about anonymity), it would not be necessary to identify them.^10^ A meeting with counsel was scheduled a month after the submission date for the report at which this matter would be further discussed (although it did not actually happen until much later).^11^

In March 2020, an application for the anonymity of Ms Moris and her children was heard by the District Judge, who declined the application (despite its not being opposed by Counsel for the U.S.). After that, it was clear what should be done, and I did indeed refer to Ms Moris and her children in my second report (August 2020) and at the subsequent hearing in the District Court (September 2020). My not naming them in my first report did not in any way affect my clinical diagnosis or prognostications. As Professor Keith Rix wrote in a report for the High Court (ruled inadmissible): ‘Professor Kopelman was going to be damned if he did and damned if he didn’t. He was in the sort of challenging position recognised by the GMC. On the one hand, he had his duty to the Court. On the other hand, he had a duty of care to the Respondent [Mr Assange], to Ms Moris, to her children and a duty to the public who have to be able to trust doctors. These were competing duties. … If the Respondent and/or Ms Moris did not want it revealed … that she was his [Mr Assange's] partner and the mother of two of his children, it would be a breach of confidentiality to reveal this information. He had a duty not to include in his report information which … if revealed to the Applicant or the public, would lead to a risk of serious harm to Ms Moris or her children or all of them …’.^12^

It was always my view that it was the job of the client, his partner, and their lawyers to determine how, when, and in what way, the existence of the relationship and the children should be revealed to the court and to the general public. That remains my view. It was not for an expert witness, commenting on clinical depression and suicidal risk, to decide this.^13^ In hindsight, I should have urged them all to come to a prompt decision about this matter, although that is not normally the role of an expert witness. Looking back, I recognise that I should have notified the Judge that there were matters that I could not yet discuss in my initial report. Whilst this may seem obvious in retrospect, it was a unique situation, completely unlike any other situation I had encountered in over 30 years of medico-legal practice, and most unlikely ever to be repeated again. At the time that my first report was being prepared and then finalised, Mr Assange's lawyers were receiving reports from 38 expert witnesses from around the world with a huge amount of information to collate before submission. Some of these reports testified about the security breaches, risks, and threats that had occurred while Mr Assange was in the Ecuadorian Embassy. Consequently, I had thought it best to seek and follow the advice of those who had overall knowledge of the situation.^14^

## 3. The hearing

In cross-examining me, Counsel for the U.S. had an obvious difficulty. I had spoken with Mr Assange on a number of occasions, whereas the psychiatric experts for the U.S. had seen him only once each, and also had a telephone call with him (during Covid). As a consequence, their reports were less thorough and well documented than mine. One of the psychiatric experts for the U.S. had, in large part, relied on my account of the background history, and the other referred to my ‘exhaustive’ history.^15^ In my view, one of these experts had given a rather selective account of the medical records, and the other had not addressed the segregated and isolated U.S. prison conditions in which Mr Assange was likely to be held, both on remand and post-conviction.

I was cross-examined for 4 hours, and the other psychiatric experts for approximately 1½ hours each. Although a searching cross-examination was to be expected in such a case, I was very surprised at the unconventional form it actually took. U.S. Counsel eschewed the most obvious way of cross-examining me but, instead, challenged my credentials and gave me a series of ‘tests’, something I had never before encountered in 30 years of cross-examination.^16^

Importantly, in the light of what followed, U.S. Counsel repeatedly challenged me about an episode in which Mr Assange had hidden a razor blade among his clothing, employing the (usual) argument that I had (allegedly) been too heavily reliant on what Mr Assange himself had told me.^17^ As I pointed out, Mr Assange had discussed this episode (and his suicidal risk) with his prison psychologist who, in consequence, had him placed back on ACCT (safety) watch, and this had been recorded in the medical records.^18^ However, this incident itself had not been properly documented elsewhere in the Belmarsh medical or ACCT records by the prison team. During lunch, Mr Assange's legal team produced further prison documentation of this episode, consisting of the record of a disciplinary hearing on this matter. Presumably anxious that his manner of questioning me on this matter might be perceived as ‘misleading’, U.S. Counsel stood up during my re-examination, addressing the District Judge: ‘Well, Madam, I was very, very particular how I put that and the transcript will show. It is in circumstances of, if it was found in circumstances which indicated a suicide risk, … Yes. I was very particular how it was put and it was not as generalised as my learned friend has put it to the professor there because we do not, I think, dispute … that some form of razor was found’.^19^ Mr Assange's Counsel said: ‘You could have fooled me, I have to say, from your questioning’.^20^ I myself said (from the witness stand): ‘Yes, that was not what I inferred this morning’.^21^ In the light of what follows, it is perhaps relevant to bear this episode in mind.

## 4. The District Court Judgment

Subsequently, the District Judge in her Judgment (January 2021) noted that I had disclosed the relationship between Mr Assange and his partner in my August 2020 report and at the hearing, and that the court had become aware of the true position in April 2020 *[i.e. following the application for anonymity]* before the reports were read or the psychiatric evidence was heard in court.^22^ She found my ‘opinion to be impartial and dispassionate’ and she was ‘given no reason to doubt [my] motives or the reliability of [my] evidence’.^23^ She ‘accepted’ the diagnoses given by myself and another psychiatrist called by Mr Assange's legal team,^24^ and she ‘preferred [our] expert opinions’ over those of the psychiatric experts called by lawyers for the U.S., giving cogent reasons for this,^25^ including the significantly less detailed summary of the medical records by one of the U.S. psychiatric experts who ‘did not appear to have access to all relevant notes’^26^ and had only ‘limited contact with Mr Assange’.^27^ In particular, she noted that I was ‘the only psychiatrist…who had assessed Mr Assange during the period May to December 2019 and was best placed to consider at first-hand his symptoms….[and had] taken great care to provide an informed account of Mr Assange's background and psychiatric history. He has given close attention to the prison medical notes and provided a detailed summary annexed to his December report’.^28^ The other psychiatrist called by the Mr Assange's lawyers was the only expert ‘with a specialism in autistic spectrum conditions’ who explained his findings ‘under robust cross-examination’.^29^ The District Judge further noted that I ‘had carefully gathered information through a series of interviews with those who knew Mr Assange well, including both parents, his current partner, and close friends and colleagues and was likely to have a fuller picture of his pre-morbid personality’.^30^ She had ‘no reason to doubt the informed and careful opinion of Professor Kopelman on this issue [suicidal risk]’.^31^

## 5. Permission to appeal

The Single Judge, the Honourable Mr Justice Swift, granted the U.S. permission to appeal on three grounds, but refused application to appeal on psychiatric grounds, stating that ‘the District Judge was entitled to rely on Professor Kopelman's evidence. It does not appear that any application was made to her to exclude Professor Kopelman's evidence. Had any such application been made on the basis set out in the Notice of Appeal, the application to exclude the evidence would have failed’. He noted that the District Judge was fully aware of the criticisms made of myself by counsel for the U.S., but had ‘nevertheless decided to accept Professor Kopelman's evidence as reliable’.^32^ The Hon. Mr Justice Swift added that further matters raised by counsel for the U.S. regarding psychiatric issues were ‘no more than an attempt to re-run determination of the evidential disputes reached by the District Judge’.^33^ I am not myself a lawyer but, in my view, this is where the matter should have rested.

## 6. The appeal process

Subsequently, Mr Justice Swift's decision was appealed before two different judges, and the psychiatric matters were brought back into the High Court hearing.^34^ One of the key points I want to make below is that, in these later hearings following the District Court Judgment, the two Counsel for the U.S. did not confine their arguments to discussing the alleged impact of my omission in not identifying Ms Moris in my first (December 2019) report. Instead, repeated aspersions were made which cast doubt on my general honesty, integrity, and character at points in the proceedings when I did not, of course, have the opportunity to respond:

### 6.1 Perfected grounds of appeal

Professor Kopelman's ‘decision to conceal’.^35^ ‘Professor Kopelman has an agenda when giving evidence’.^36^ ‘Professor Kopelman had concealed’.^37^ ‘Professor Kopelman's motivation may have been a desire to play the system’.^38^

### 6.2 Renewed grounds of appeal

‘The Judge was wrong in law, not to treat the evidence as incapable of being relied upon because of Professor Kopelman's deceit’.^39^ ‘If what he says is true…’.^40^ ‘Professor Kopelman deliberately suppressed’.^41^ ‘He still suppressed the detail until access to his hand-written notes …’,^42^ ‘… cast doubt upon his impartiality and credibility’.^43^

### 6.3 Transcript of appeal pre-hearing

Junior Counsel for the U.S., also a K.C.,^44^ used the word ‘mislead’ or ‘misleading’ 24 times, and ‘duty’ (as in ‘to the court’) 15 times, clearly indicating the impression she wished to convey about me. For example: ‘He was willing to subjugate his duty to the Court’.^45^ ‘…willing to mislead …’.^46^ ‘He has failed in his duty’.^47^ ‘The misleading nature of his first report’.^48^ ‘Professor Kopelman had been willing to mislead’.^49^

### 6.4 U.S. Government skeleton argument for appeal

‘His lack of independence rendered his evidence inadmissible’.^50^ ‘His willingness to mislead her [the District Judge] impacted upon the overall reliability of his evidence’.^51^ ‘Professor Kopelman deliberately set out to misrepresent the position’.^52^ ‘Kopelman deliberately suppressed in his report a highly relevant factor to the question of likelihood to suicide’.^53^ ‘Asides his willingness to mislead, Professor Kopelman's approach to diagnosis gave cause for concern [*See below, 7. Re-litigation of issues, 7.1 ICD*]. Asides his willingness to mislead …’.^54^ ‘The District Judge should have decided that she could not rely upon Professor Kopelman's evidence at all or ought to have attributed far less weight to it’.^55^

### 6.5 Appeal hearing

U.S. Counsel used ‘mislead’ or ‘misleading’ 18 times. For example: ‘That is germane to the fact that Professor Kopelman was misleading …’.^56^ ‘It was conveying the clear impression that these were not Mr Assange's children. Now, that is not in dispute because Professor Kopelman accepted that is what he wanted to achieve’.^57^ [-*U.S. counsel's interpretation, not mine*]. ‘No weight should have been given to Professor Kopelman’.^58^ ‘…no weight or the inadmissibility of Professor Kopelman …’.^59^ ‘Professor Kopelman misleading the Court…’.^60^ ‘Professor Kopelman had been willing to mislead on a material issue’.^61^ ‘Professor Kopelman was either not admissible or should have been given no or considerably less weight … difficult to judge the overall reliability of his opinions when we see his evidence as a whole’.^62^

### 6.6 Summary

These comments should be seen in the light of the District Judge's and the Hon Mr Justice Swift's Judgments. However, they also raise the very important question of when does legitimate criticism become pejorative characterisation of character, integrity and competence (*see below: 11.3 Where are the boundaries?*).^63^

## 7. Re-litigation of issues determined by District Judge

### 7.1 International Classification of Diseases (ICD)

In addition to all this, U.S. Counsel also made aspersive comments in the High Court about my evidence relating to other matters on which the District Judge had already ruled. These were presumably to cast further doubt on my professional competence and credibility and, again, these occurred at stages in the proceedings when I did not myself have the opportunity to respond. U.S. Counsel claimed that my ‘approach to diagnosis gave cause for concern…By dismissing the objective criteria by which psychiatrists judge mental illness’,^64^ and that I did not have ‘familiarity with the ICD (International Classification of Diseases)’.^65^ On this matter, U.S. Counsel cannot have read my reports with appropriate attention: I had given ICD diagnoses and their codings for each and every diagnosis I made*.* In my first Report, ICD-10 diagnoses and codings were given 10 times between pages 29 and 34. On pp. 30/31, I had spelled out the ICD criteria which Mr Assange fulfilled for a ‘severe’ depressive episode. In my second report, ICD diagnoses and codings were given 6 times between p.14 and p. 19*.* By contrast, the psychiatrists for the U.S. did not do this, nor did they explicitly spell out their diagnostic criteria. I also held up in court the Institute of Psychiatry ‘*Notes on eliciting and recording clinical information’ (Oxford Medical Publications),* of which I myself have written a succinct account,^66^ to indicate the manner by which I take a clinical history.^67^ The District Judge had heard and observed all this. The question, therefore, arises as to why was Counsel for the U.S. permitted to reiterate and re-litigate this spurious argument in the High Court?

### 7.2 Summary of the medical records

Likewise, U.S. Counsel kept claiming that ‘Mr *[sic]* Kopelman's psychiatric opinion was contingent upon the self-report of Mr Assange in a large part, and one of the key differences between his approach and that of [the psychiatrists] for the Appellant is that they considered the long running day-to-day notes and observations made by the clinical staff and other staff at Belmarsh did not support, or were inconsistent, with some of Professor Kopelman's findings’,^68^ and ‘… they considered the long running day-to-day notes made by staff and medical practitioners to be important in discerning the true picture’.^69^ Again, this was re-litigating a point that had been determined in the District Court, where this issue had been very fully discussed. I had provided 13 small print pages summarising the medical records in my first report, and a further 5¼ small print pages in my second report. In summarising the medical records, the psychiatrists, called by the U.S., provided only 4½ pages and 3½ (large font) pages, respectively, and one of these accounts was particularly selective. The District Judge had determined: ‘[This psychiatrist's] summary of the notes was significantly less detailed than the summary provided by Prof Kopelman, and he did not appear to have access to all the relevant notes…He had not read the notes from his [Mr Assange's] admission to Healthcare^70^…[The other psychiatrist, called by the U.S.] did not dispute that Mr Assange's depression was severe at the time he was assessed in 2019’.^71^ Again, why was U.S. Counsel allowed to raise and re-litigate this matter in later proceedings, when I was not permitted to respond?

### 7.3 The Meltzer report

Similarly, another matter of re-litigation raised during the Appeal^72^ concerned the report of Mr Nils Meltzer, UN Special Rapporteur on Torture and other Cruel, Inhuman or Degrading Treatment or Punishment. He had visited Mr Assange at the Ecuadorian Embassy, accompanied by two doctors, one a psychiatrist. At the District Court hearing, U.S. Counsel expressed strong disapproval of Mr Meltzer's report, which he described as ‘neither balanced nor accurate’^73^ and ‘biased and unobjective’.^74^ He had quoted to me a series of political statements^75^ from the report, as if I had somehow sanctioned them. However, I had included in my own report only those observations from the Meltzer report which were medical or psychiatric (i.e. from the accompanying doctors), as I explained repeatedly at cross-examination in the District Court.^76^ U.S. Counsel's quotations were political comments, which I had very deliberately omitted from my reports, as I pointed out in cross-examination (again stated several times^77^). Regardless, U.S. Counsel had continued to quote these political statements to me in the District Court, and he appeared to me to be somewhat floundering at this point. Nevertheless, he was allowed by Their Lordships to raise this matter again at appeal.^78^ Why?

## 8. Privacy and confidentiality

Another matter of concern from this case relates to the court's approach to sensitive matters of privacy and confidentiality. When U.S. Counsel cross-examined me in the District Court on my not having identified Ms Moris in my first report, I stated that I had sought advice from instructing lawyers on what to do in this unique situation^79^ (*see above: 2. The issue of anonymity*). Soon after this, U.S. Counsel asked me why I had also not included in my report something concerning the private life of Mr Assange's eldest (adult) son that I had recorded in my hand-written notes. U.S. Counsel simply blurted the matter out,^80^ claiming that I had not pursued an apparent inconsistency between Mr Assange's and his partner's accounts of this. My response was that the matter concerned the son's private life and was of no relevance whatsoever to Mr Assange's case^81^ (see *GMC*^82^ *guidelines on confidentiality*^83^ which would have precluded my introducing this matter). At the High Court, U.S. Counsel summarised my response as ‘he wriggles’^84^ although he did not quote my response in its complete fullness.^85^ U.S. Counsel^86^ and Their Lordships in their Judgment (December 2021)^87^ both pored over my statements in response to that part of the cross-examination. Had I been asked what I was thinking at that point of the cross-examination, I would have said that I was greatly shocked and surprised by the fact that U.S. Counsel was paying no regard to the safety and security of young children, nor the privacy of a non-participant in the hearing. This issue perhaps illustrates an important cultural difference between legal and medical professional expectations and ethos, as Prof Rix had discussed in his report^88^ (*see below: 11.7: Wider issues in court*).

More specifically, at one point in the appeal against the Single Judge's decision, Junior Counsel for the U.S. (herself a KC^89^) referred to my response in the District Court, when I had been repeatedly challenged about my being a neuropsychiatrist and general psychiatrist rather than a forensic psychiatrist. She described my response as not being what ‘might be expected’.^90^ She declined to read out what I had said, because it was ‘rather…personal in respect of [U.S. Counsel]’.^91^ Instead, she simply drew Their Lordships’ attention to the written description of what I had said at the point indicated. This was: ‘I have given evidence in court on many occasions for the last 30 years and indeed we *[i.e. U.S. Counsel and I]* did the *Tollman* case together and, since that time, I have been rung up and emailed by solicitors on about five or six occasions, telling me that there is an extradition case in Wandsworth, [*U.S. Counsel*] is the counsel, and he is very keen to have your services, so I think it is a bit rich, Mr_____, if you challenge my credentials’.^92^ The District Judge laughed at this point, and asked U.S. Counsel: ‘How would you like to respond that, Mr_____?’^93^ He replied: ‘I cannot respond other than *[addressing me]* you are obviously, we might say that you are more of an advocate than a psychiatrist’, to which I said, ‘I want to use an unparliamentary word to that’.^94^ Hence, I was roundly criticised *[see next paragraph]* for attempting to protect the safety and security of infants; and U.S. Counsel showed no compunction in raising in court private but irrelevant matters from the life of Mr Assange's eldest son. But when it came to sparing U.S. Counsel's own blushes, his colleague thought that this was a matter which should be ‘deliberately suppressed’ *[my words]* in court. To avoid the appearance of double standards, perhaps notions of privacy need to be applied more evenly in future between members of the legal profession and other parties in court?

## 9. High Court Judgment

In their Judgment of 10 December 2021, Their Lordships devoted 22 paragraphs to re-examining the issue of my not having identified the partner and children in my first (pre-anonymity hearing) report, stating that Professor Kopelman was not ‘confronted with a dilemma of such difficulty as has been claimed’.^95^ Despite the previous Judgments,^96,97^ they appeared wholly to adopt U.S. Counsel's views, ignoring the carefully reasoned account by Mr Assange's Counsel of what had actually occurred,^98,99^ and seeming to disbelieve that I had sought legal advice in this unique situation^100^ and that my initial caution in disclosing the full family position was the result of this discussion.^101^ Their Lordships also argued that an application under Rule 19.9^102^ of the Criminal Procedure rules could have been made.^103^ Had the anonymity hearing been held much earlier than in March 2020, I would not have been placed in this difficulty, and this fact was indeed discussed at both the pre-hearing for the appeal^104^ and again at the actual Appeal,^105^ but this was never mentioned in the High Court Judgment itself. Instead, Their Lordships noted that the District Judge had learned of Mr Assange's relationship with his partner ‘before she read the medical evidence: it was no thanks to Professor Kopelman that she had done so’.^106^ But Their Lordships did not mention here that she had learned of this at the application for anonymity in March 2020, brought quite properly by lawyers for Mr Assange after discussion with the client and his partner and, interestingly, unopposed by lawyers for the United States (*see my comments above: 2. The issue of anonymity, final paragraph*)*.*

## 10. Press reports

Despite Mr Assange's Counsel having expressed alarm in court at inaccurate press reports,^107^ such inaccuracies continued. One newspaper reported that I had ‘withheld evidence from Julian Assange's first extradition hearing’ and claimed that I had, thereby, ‘misled’ the District Judge on the matter. In fact, the existence of a partner was acknowledged in my first report, and Mr Assange's relationship with Ms Moris and their children had been clearly stated in my second report (following the unsuccessful application for anonymity), and it was openly discussed at the actual hearing. Nothing at all was withheld from the extradition hearing, as the District Judge's Judgment confirmed. However, when I consulted two sets of libel lawyers about this, I was advised that the press would ‘take cover’ under what the legal team for the U.S. had said about me.

## 11. Discussion and conclusions

### 11.1 General issues for expert witnesses

This case raises a number of important issues regarding the role of expert witnesses in court which, I suggest, require much fuller discussion than they have received to date. However, it is perhaps important, first, to acknowledge some background factors, applicable to all psychiatric/psychological reports. There is occasionally a potential conflict between a person's duties as an expert witness versus his/her duties as a doctor or clinician, particularly with respect to confidentiality.^108^ As highlighted by Prof Rix in his report to the court,^109^ this arose in the present case with respect to non-participants: the naming of Mr Assange's partner, his children and their safeguarding, and the privacy of Mr Assange's eldest son. But it can occur in other cases with respect to incriminating disclosures during the course of a psychiatric interview. It is, of course, paramount that the expert provides his/her honest and independent opinion to assist the court, regardless of whoever has instructed him/her;^110,111^ very occasionally (in my experience) undue pressure is applied by either the instructing lawyer, the defendant, and/or his/her family.^112^ That was not the case here. Finally, it is, of course, a common cross-examination ploy to argue that an expert has relied too much on a defendant's/respondent's (‘hearsay’) account: such an account needs to be framed within a thorough clinical history and mental state assessment, corroborated, as here, by appropriate clinical rating scales, psychometric and performance validity tests, detailed and unbiased summaries from the Inmate Medical Records, informant reports, and spelled-out diagnostic criteria.^113,114^ In the present reports, there was also an exceptionally large number of citations to the relevant clinical and scientific literature.

### 11.2 Legal versus medical decisions

On more specific matters relating to the present case, the first concerns what is a legal responsibility and what is a medical duty. The timing of the anonymity application, and the decision about when/how to identify the partner and children, were surely not medical decisions to make (or not solely medical decisions), but this was never stated, or even considered, in Their Lordships’ Judgment. As for Section 19.9 (*see above: 9. High Court Judgment*), this had been introduced to the Criminal Procedure rules (1^st^ April, 2019) only 8 months before I submitted my first report, and this was surely a legal, not a medical, matter?

### 11.3 Where are the boundaries?

My error of omission (which is what it was) concerned the identification of the partner and youngest two children in my initial report only, although the existence of a caring partner had been clearly acknowledged there.^115^ In hindsight, I should have pressed the client, his partner, and his lawyers to sort out the anonymity issue before I was due to submit my first report, but that is not normally the role of an expert. When this did not happen, I should have notified the Judge that there were matters I did not wish to include in my report for what were then thought (with good reason) to be risks to the safety and security of the partner and children. However, these were unique circumstances, and it had seemed to me entirely reasonable to consult an instructing (and very experienced) lawyer about what to do in such an unusual situation. But, should the lawyers for the U.S. have been allowed to go beyond this in making repeated and generalised, disparaging statements about my ‘deceit’, credibility, and reliability – matters of character and reputation? The clear purpose of these aspersions was to have my evidence to the District Court downgraded in terms of admissibility or weight,^116^ but when does legitimate cross-examination/criticism become derogatory characterisation of integrity and competence? After the District Judge and the Single Judge had ruled on these matters, why were the lawyers for the U.S. allowed by the High Court to repeat these pejorative aspersions over and over again (*see above: 6. The appeal process*), and to re-litigate arguments which had failed in the District Court? (Compare: Mr Assange's Counsel;^117,118^ and Swift^119^).

### 11.4 False press reports

In particular, should it not be a concern that the U.S. lawyers were allowed to make statements which allegedly gave ‘cover’ to (potentially) libellous statements in the press, where it was falsely claimed that I had withheld information at the District Court hearing? The U.S. lawyers will point out that they themselves did not actually make this claim, but should not the High Court have stepped in and asserted more control over what U.S. counsel was allowed to say about me in order to prevent potentially libellous inferences being made – especially after the Mr Assange's Counsel had warned the court of this risk?^120^

### 11.5 Opportunity to respond

Moreover, the repeated iterations of my being ‘misleading’ came in the context of U.S. counsel's own debatable manner of questioning me about the razor blade (*see above: 3. The hearing, final paragraph*), and the distortions and inaccuracies concerning what I had said about ICD diagnosis, ‘malingering’ tests,^121^ the medical records, and the UN Rapporteur's report. The District Judge had ruled in my favour on these matters in her Judgment; and Their Lordships ultimately agreed that the District Judge had heard the psychiatric evidence and that, therefore, her Judgment on this must stand. Why was U.S. Counsel, nevertheless, allowed to repeat and re-litigate his damaging claims about me at each stage in the subsequent proceedings, when I could not personally respond? The Supreme Court of Western Australia has recently ruled that ‘Judges are not entitled to criticise expert witnesses by reference to expert material not in evidence without those witnesses having an opportunity to respond’.^122^ Should not a similar rule apply here?

### 11.6 The use and misuse of ICD and DSM

There is a particular point to be made about ICD (the International Classification of Diseases) which applies to other cases as well. As mentioned above, I had cited the appropriate ICD diagnoses throughout my reports (unlike the psychiatrists for the U.S.), and I had specified the ICD criteria which Mr Assange fulfilled in terms of a ‘severe’ depressive episode. But ICD is a reference volume (like Archbold), not a psychiatric textbook. I quoted in the District Court from the introduction to the rival ‘Diagnostic and Statistical Manual of Mental Disorders’ (DSM) on this matter: ‘The diagnostic categories, criteria and textual descriptions are meant to be employed by individuals with appropriate clinical training and experience in diagnosis. … The specific diagnostic criteria … are meant to serve as guidelines to be informed by clinical judgement and not meant to be used in a cookbook fashion’.^123^ In other words, these volumes (ICD/DSM) are guidelines to be used by appropriate clinical experts; they are not ‘cookbooks’ from which a lawyer may advocate a diagnosis or adduce the level of severity of a clinical condition, which is what counsel for the U.S. was attempting to do in the present case. There are various reasons why the understanding and use of ICD requires the context of clinical evaluation; and I myself have published critiques of ICD-10 & ICD-11 (Kopelman and Fleminger, 2002; Kopelman, 2005, 2022),^124^ and sat on two committees advising on ICD-11. This was a matter beyond U.S. Counsel's expertise, as no doubt the District Judge had observed. Once again, why was U.S. Counsel allowed to raise and re-litigate this matter in the subsequent High Court hearings, when the sole purpose appeared to be to cast doubt on my professional competence and credibility, and I was not myself in a position to respond?

### 11.7 Wider issues in court

There are other issues to be considered. Their Lordships stated I had not been ‘confronted with a dilemma of such difficulty’, and their Judgment appeared to imply that the only considerations which count in court are legal ones. I fully accept that legal considerations should have primacy and, once the anonymity issue had been resolved, I gave them full primacy. But, as Prof Rix had argued (*see above: 2. The issue of anonymity, penultimate paragraph*),^125^ there can be ‘competing duties’, and this overriding duty should not ‘ride roughshod’ over all other duties as a doctor. I was very much aware of these competing professional obligations when I prepared my initial report, which is why I had sought advice on the matter. Mandatory trainings on infant and childhood safety have become commonplace in medicine. Some academic lawyers^126^ have argued that it is time for greater training for lawyers on psychological matters,^127^ including on the safeguarding of children,^128^ unconscious and conscious bias (including in adjudicators),^129,130^ and the vulnerability of witnesses in rape and sexual assault cases.^131^ The Children and Families Act, 2014,^132^ has made children's interests paramount in Family Courts. Perhaps it is time that the adult courts were brought into line with the ethos of other professional disciplines on this matter, and that psychological sensitivities, as well as legal niceties, were fully acknowledged in adult courts?

### 11.8 Courtesy and proportion

There is also the question of good manners. An eminent U.S. Attorney, Eric Lewis, giving evidence on behalf of the Respondent (Mr Assange), said to U.S. Counsel cross-examining: ‘I have been courteous to you, I would ask that you be courteous to me’.^133^ Mr John Pilger in his blog (dated 16 August, 2021)^134^wrote: ‘Watching the lead barrister acting for Washington… smearing witnesses, especially Kopelman …’. As a 71-year-old senior member of my profession, I expected the 63-year-old Lord Chief Justice to adjudicate on matters of law (such as the U.S. proposed ‘assurances’^135^), not to make snide comments (‘no thanks to Professor Kopelman’^136^). As a psychiatrist and psychologist, I cannot help but wonder why the High Court Judgment spent a whole 22 paragraphs deriding me on matters that had already been dealt with by the District Judge and the Single Judge regarding a set of circumstances that are most unlikely ever to be repeated, whilst ultimately accepting the District Judge's decision on Mr Assange's mental health. Their Lordships then determined that, had the U.S.'s newly presented [to the High Court] ‘assurances’ been put to the District Judge, she would have decided differently on extradition.^137^ Those 22 paragraphs stand in very striking contrast to the single sentence in this Judgment which Mr Assange's Counsel described as constituting ‘the entirety of the High Court's substantive consideration of the two pivotal [U.S.] assurances in the present case’.^138,139^

### 11.9 Recruitment of experts

As mentioned at the outset, I have long been concerned about the recruitment of appropriate experts into the courts.^140^ Changes in NHS requirements, university funding, and Legal Aid have made this increasingly difficult, particularly in criminal or extradition cases. But what inhibits many senior scientists from putting themselves forward is concern about what might happen under cross-examination. Often, such concerns are misplaced (I have long thought that being an academic is a good training for being a court expert), but gratuitous ill manners, distortion and misrepresentation of what an expert has said, not to mention pejorative aspersions repeated over and over again at stages in legal proceedings when the expert cannot respond – none of this will encourage potential experts to put themselves forward in future. Moreover, when things go wrong, as they will do from time to time, it may be the consequence, as here, of a number of interacting factors – the delayed anonymity hearing, conflicting professional obligations, legal advice which was (in hindsight) unwise but well meaning, and the expert's decision to follow that advice. Behavioural scientists are well used to having to tease apart such complex interactions. If courts want to recruit more and better experts, and to retain and protect them, perhaps a more nuanced response, or some (non-personalised) notion of ‘collective responsibility’, might be a fairer reaction to such situations, rather than the knee-jerk reaction of placing all blame on an expert witness?

### 11.10 Conclusion

Finally, and most importantly, I had given the court my honest and considered opinion on Mr Assange's clinical diagnosis and suicidal risk, were extradition to be ordered. Others might disagree with my opinion, but I profoundly object if a lawyer chooses to question my honesty and integrity on this issue. My message may have been unpalatable to some, but surely it should not justify ‘shooting the messenger’?^141^

## Notes

 1. See Reference 1.

 2. See Reference 2.

 3. USA v. Stanley and Beatrice Tollman, 2008, EWHC 184 (Admin).

 4. USA v Ian Norris, 2009, EWHC 995 (Admin); 2010, UKSC 9.

 5. South Africa v. Shrien Dewani, Westminster Magistrates Court, 10 August, 2011 and 2012 EWHC 842 (Admin).

 6. USA. v. Lauri Love, 2018, EWHC 172 (Admin).

 7. USA. v. Lauri Love, 2018, EWHC 172 (Admin).

 8. Prof Kopelman's medico-legal CV is available on email request to the author.

 9. As corroborated in the Skeleton Argument for Appeal on behalf of the Respondent, pp. 10-11, paras 3.2-3.3; p. 13, paras (iii)–(vi); the High Court transcript, Day 1, page 88, para F; and the High Court transcript, Day 2, p. 28, E/F; p. 30, paras A–F; pp. 30-31, G-A.; pp. 33-34, paras H-A. The District Judge was also aware of this sequence of events: see Note 14, below.

10. See above.

11. Skeleton Argument for Appeal on behalf of the Respondent, p. 11, para 3.3, and p. 13, para (vi).

12. Prof Keith Rix report, pp. 63-64, paras 7.1 & 7.3.

13. That view was fully supported by Mr Assange's legal team, although it was not seemingly upheld by the High Court; see below: *11. Discussion and conclusions, 11.2 Legal versus medical decisions*.

14. The District Judge had been fully aware of this sequence of events (Transcript of my testimony at the District Court at p. 57, ll.29-32, and District Court Judgment, Westminster Magistrates’ Court, USA v. Julian Paul Assange, 4 Jan 2021, para. 330), and the High Court was also given a very full account by Mr Assange's lawyers – see Note 9, above.

15. At para 3.1, and p. 3, para 11, of their respective reports.

16. For example, I was asked what were the last two papers I had read on ‘malingering’; I could not remember in the witness box, although I regularly review papers for scientific journals on what are properly called Performance Validity Tests. Later in the day, it emerged that U.S. Counsel's own knowledge about performance validity tests was itself very limited (he claimed that the TOMM was not a ‘malingering’ test, apparently unaware that it stood for Test of Memory Malingering). More relevantly, neither of U.S. Counsel's psychiatric experts had raised or discussed malingering in their reports, calling into question the pertinence of this line of questioning.

17. Transcript of my testimony at the District Court, p. 27, ll. 6–8.

18. Transcript of my testimony at the District Court, p. 26, ll. 29–31.

19. Ibid, page 80, lines 3–12.

20. Ibid, page 80, lines 3–12.

21. Ibid, page 80, lines 3–12.

22. District Court Judgment, Westminster Magistrates’ Court, USA v. Julian Paul Assange, 4 Jan 2021, para. 330.

23. Ibid, para. 331.

24. Ibid, paras 332–333.

25. Ibid, paras 334–336.

26. Ibid, para 336.

27. Ibid, para 335.

28. Ibid, para 332.

29. Ibid, para 333.

30. Ibid, para 335.

31. Ibid, para 349.

32. High Court of Justice, [2021] QB (Admin), CO-150-2021, pages 1–2, para 2.

33. Ibid, p. 2, para 3.

34. USA v Assange [2021] EWHC 2528 (Admin) (11 August 2021).

35. Perfected Grounds of Appeal, page 10, para 330.

36. Ibid, page 28, para 95.

37. Ibid, page 28, para 97.

38. Ibid, page 29, para 98.

39. Renewed Grounds of Appeal, page 211, para 3.

40. Ibid, page 213, para 405.

41. Ibid, page 213, para 405.

42. Ibid, page 213, para 405.

43. Ibid, page 214, para 6.

44. QC at that time.

45. Transcript of Appeal Pre-hearing, p. 14, line 2.

46. Ibid, p. 14, l.4.

47. Ibid, p. 17, B.

48. Ibid, p. 18, l.2.

49. Ibid, p. 14, l. 4.

50. Skeleton Argument for Appeal, U.S. Government, p. 1, para 5, bottom line.

51. Ibid, p. 2, para 5, l.4.

52. Ibid, para 86.

53. Ibid, para 87.

54. Ibid, para 90.

55. Ibid, para 102.

56. High Court transcript, Day 1, page 49C.

57. Ibid, page 50B.

58. Ibid, page 52G.

59. Ibid, page 60C.

60. Ibid, page 81G.

61. Ibid, page 83F.

62. Ibid, page 86 G/H.

63. The clear purpose of these aspersions was to have my evidence to the District Court downgraded in terms of admissibility or weight – High Court transcript, Day 1, p. 48C – as had been anticipated by the Hon Mr Justice Swift (*see above**: 5. Permission to appea**l*). At one point during my cross-examination in the District Court, Counsel for the U.S. had asked ‘In fact, Professor, it is in fact the fact that someone has got a partner and children is what is described as by psychiatrists as the best known protective factors against suicide?’ – Transcript of my testimony at the District Court, p. 58, ll. 6–7 – but that point had clearly been acknowledged in the report he was criticising – ‘In London, Mr Assange has various protective factors available to him,…He speaks to his partner by telephone nearly everyday. She also visits him. He has been visited by some of his children,…’ (my Report of December 2019, p. 34, Opinion para 11, ll. 1–4).

64. Skeleton Argument for Appeal, U.S. Government, paragraphs 90–91.

65. Ibid, para 93.

66. See Reference 3 and Reference 4.

67. When I mentioned ‘an old-fashioned Maudsley history’ in the transcript of my testimony at the District Court at p. 5, l. 11.

68. High Court transcript, Day 1, page 48E.

69. Skeleton Argument for Appeal, U.S. Government, page 26, para 92.

70. District Court Judgment, Westminster Magistrates’ Court, USA v. Julian Paul Assange, 4 Jan 2021, para. 336.

71. Ibid, para 336.

72. High Court transcript, Day 1, page 70, paras E–H.

73. Transcript of my testimony at the District Court, p. 62, l. 18.

74. Ibid, p. 65, l.31.

75. In his book, *The Trial of Julian Assange* (Virgo, 2023), Nils Meltzer points out that these statements were actually ‘legal’ rather than ‘political’, the word I used in court. The point here is that they were non-medical and, consequently, I had deliberately omitted them from my report.

76. Transcript of my testimony at the District Court, for example at p. 57, ll.29–32, p. 58, ll. 26–28.

77. Ibid, p. 57, ll.29–32, p. 58, ll. 26–28.

78. High Court transcript, Day 1, pages 70-71, paras E-H & A.

79. Transcript of my testimony at the District Court, p. 57, ll. 29–32, p. 58, ll. 26–28.

80. This may have been inadvertent, but all the more reason why Counsel should not have been questioning me about the private life of a (non-participant) family member.

81. Transcript of my testimony at the District Court, p. 62, ll. 8–10.

82. General Medical Council.

83. GMC: Confidentiality: Good practice in handling patient information, page 40, para 91. See Reference 5.

84. High Court transcript, Day 1, p. 70 para B.

85. High Court transcript, Day 1, p. 62, ll. 8–10.

86. Skeleton Argument for Appeal, U.S. Government, p. 23.

87. USA vs Julian Paul Assange, [2021] EWHC 3313 (Admin), para 79.

88. Prof Keith Rix report, p. 56, para 6.3.5.

89. QC at that time.

90. Transcript of Appeal Pre-hearing, p. 11, para C/D.

91. Ibid, p. 11, para C/D.

92. Transcript of my testimony at the District Court, p. 1024, ll. 1–10.

93. Transcript of my testimony at the District Court, p. 1024, ll. 1–10.

94. Ibid, p. 1024, ll. 1–10.

95. USA vs Julian Paul Assange, [2021] EWHC 3313 (Admin), para 86.

96. District Court Judgment, Westminster Magistrates’ Court, USA v. Julian Paul Assange, 4 Jan 2021.

97. High Court of Justice, [2021] QB (Admin), CO-150-2021.

98. Skeleton Argument for Appeal on behalf of the Respondent, pp. 10-11, paras 3.2–3.3; p. 13, paras (iii)–(vi); High Court transcript, Day 1, page 88, para F; High Court transcript, Day 2, p. 28, E/F; p. 30, paras A–F; pp. 30–31, G–A.; pp. 33–34, paras H–A.

99. In the Skeleton Argument for the Appeal and during the High Court appeal itself, Mr Assange's lawyers provided a full chronology of events, and drew attention to the ‘unique and special’ circumstances in which there were ‘real concerns’ about the risk to Ms Moris and her children, expanding upon the evidence for this risk. They corroborated that I had sought legal advice in this unprecedented situation, and that the position was disclosed at the anonymity hearing in March, and openly discussed in my second (August) report and at the hearing in September. Moreover, the District Judge (aware of all this) had found my ‘opinion to be impartial and dispassionate’ and had not doubted the reliability of my evidence.

100. USA vs Julian Paul Assange, [2021] EWHC 3313 (Admin), para 86.

101. Skeleton Argument for Appeal on behalf of the Respondent, p. 13, para (iv).

102. A legal application to withhold information in the public interest.

103. USA vs Julian Paul Assange, [2021] EWHC 3313 (Admin), para 87.

104. Transcript of Appeal Pre-hearing, p. 32D.

105. High Court transcript, Day 2, p. 34D.

106. USA vs Julian Paul Assange, [2021] EWHC 3313 (Admin), para 89.

107. High Court transcript, Day 2, p. 26, D–F.

108. See Reference 6, pp. 8–11.

109. Prof Keith Rix report, pp. 63-64, paras 7.1 & 7.3.

110. See Reference 7, pp. 533, 538–545.

111. See Reference 6, pp. 4–10.

112. This has happened to me on a few occasions in my career, in each instance because I had refused to make the diagnosis or inference that the instructing lawyer/defendant/family member wanted me to make.

113. See above: *4 District Court Judgment, and also 7. Re-litigaton of issues, 7.1 ICD*.

114. See Reference 6 and Reference 7 for fuller discussion.

115. See Note 63, above.

116. See High Court transcript, Day 1, p. 48C. See also comments by the Hon Mr Justice Swift (above, *5. Permission to appeal*).

117. Skeleton Argument for Appeal on behalf of the Respondent, p. 4, para 1.11.

118. High Court transcript, Day 2, p. 44, para C.

119. High Court of Justice, QB (Admin), CO-150-2021, page 2, para 3.

120. High Court transcript, Day 2, p. 26, D–F.

121. See Note 16, above.

122. *Moyes v Ensco Australia Pty Ltd* [2022] WASCA 104.

123. DSM-IV-TR, p. xxxii.

124. See References 8–10.

125. Prof Keith Rix report, p. 56, para 6.3.5.

126. See Reference 11.

127. Ibid.

128. See Reference 12.

129. See Reference 11.

130. See Reference 2.

131. See Reference 13. See also: C. Proudman, *The Guardian* 18 December 2024.

132. Children's and Families Act, 2014, https://www.legislation.gov.uk/ukpga/2014/6/contents.

133. USA v. Assange Core Permission Bundle (complete transcripts of District Court hearing), p. 921, ll. 27–28.

134. See http://johnpilger.com/articles/a-day-in-the-death-of-british-justice.

135. In this case, lawyers for the U.S. had presented four ‘assurances’ to the High Court regarding the conditions of Mr Assange's incarceration, should he be extradited.

136. See also above: *9. High Court Judgment*, and Note 106.

137. USA vs Julian Paul Assange, [2021] EWHC 3313 (Admin), paras 61 and 99.

138. Application for Leave to Appeal to the Supreme Court, Form 1, para 17.

139. The very unusual social and political context in which these hearings were held is perhaps illustrated by the presence in the U.K. court of American lawyers and Australian officials (including, at times, the Australian High Commissioner to the U.K.). This background social and political context is further illustrated by a *Washington Post* article (Reference 14), summarising internal disagreements within the U.S. Justice Department, and the manoeuvres that ultimately resulted in the plea bargain: https://www.washingtonpost.com/national-security/2024/06/27/assange-wikileaks-deal-justice/.

140. See Reference 1.

141. As a result of the District Judge's decision being overturned, Mr Assange spent a further 3 years in prison, and the case was still unresolved at the time of the plea bargain. It is perhaps time that the appropriate role of psychiatric evidence in extradition courts should be reviewed, and also whether there should be such a narrow emphasis on the estimated degree of suicidal risk (‘high’, ‘very high’, etc.) in extradition cases (see also Reference 15, p. 46), as opposed to (for example) the risk of psychological damage from being placed in long-term segregation/isolation – but that is a debate for another occasion.
